# Hematologic malignancies in South Africa 2000–2006: analysis of data reported to the National Cancer Registry

**DOI:** 10.1002/cam4.597

**Published:** 2016-01-15

**Authors:** Sara J. Schonfeld, Friederike Erdmann, Tracey Wiggill, Elvira Singh, Patricia Kellett, Chantal Babb, Joachim Schüz

**Affiliations:** ^1^ Section of Environment and RadiationInternational Agency for Research on Cancer (IARC)LyonFrance; ^2^ Department of Haematology and Molecular MedicineNational Health Laboratory ServiceJohannesburgSouth Africa; ^3^ National Cancer RegistryNational Health Laboratory ServiceJohannesburgSouth Africa; ^4^ Division of Human GeneticsFaculty of Health SciencesUniversity of the WitwatersrandJohannesburgSouth Africa

**Keywords:** Hematologic neoplasms, leukemia, lymphoma, population groups, South Africa

## Abstract

Little is known about the incidence patterns of hematologic malignancies in Sub‐Saharan Africa, including South Africa. We estimated incidence rates of pathology‐confirmed adult cases of leukemia, myeloma and related diseases (myeloma), Hodgkin lymphoma (HL), and non‐Hodgkin lymphoma (NHL) reported to the National Cancer Registry of South Africa (NCR) between 2000 and2006, by age, gender, and population group (Black, White, Coloured, Asian/Indian). Gender‐specific age‐standardized rates were calculated overall and by population group and incidence rate ratios (IRRs) were estimated using Poisson regression models. Between 2000 and 2006, there were 14662 cases of leukemia, myeloma, HL, and NHL reported to the registry. Incidence rates of reported hematologic malignancies were generally 20–50% higher among males than females. Our analyses suggested marked differences in the rates of reported hematologic malignancies by population group which were most pronounced when comparing the White versus Black population groups (IRRs ranging from 1.6 for myeloma to 3.8 for HL for males and females combined). Challenges related to diagnosis and reporting of cancers may play a role in the patterns observed by population group while the set‐up of the NCR (pathology‐based) could lead to some degree of under‐ascertainment in all groups. This is the first country‐wide report of the incidence of hematologic malignancies in South Africa. Despite challenges, it is important to analyze and report available national cancer incidence data to raise awareness of the cancer burden and to characterize patterns by demographic characteristics so as ultimately to improve the provision of cancer‐related health care.

## Introduction

Worldwide, leukemia, multiple myeloma (MM), Hodgkin lymphoma (HL), and non‐Hodgkin lymphoma (NHL) collectively accounted for an estimated 6.5% of new cancer cases in 2012 with the majority of these cases coming from NHL followed by leukemia 1. While global estimates suggest two and threefold higher incidence rates of NHL and leukemia, respectively, in high income countries compared to Sub‐Saharan Africa 1, there is little known about the incidence patterns of hematologic malignancies in this region, including South Africa. Similar to worldwide figures, hematologic malignancies were estimated to contribute about 6% of new cancer cases in South Africa in 2012 1.

To date, the literature of hematologic malignancies in South Africa is largely based on hospital‐based studies which report on patient and disease characteristics of leukemias and lymphomas 2, 3, 4, 5, 6, with a particular focus on the prevalence of HIV and the differences in cancer characteristics between HIV‐positive and ‐negative patients. There is a well‐established association between HIV and several types of hematologic malignancies, including but not restricted to the AIDS‐defining subtypes of NHL 7, 8, 9. While hospital‐based studies benefit from detailed patient information, there is also a need to estimate incidence and mortality rates, particularly at the national‐level. Such data provide important information about the overall burden of disease, which in turn inform cancer control strategies, and provide a basis for investigating underlying determinants of disease.

Studies from the United Kingdom and United States show considerable variability in the incidence of hematologic malignancies by gender and age 10, 11, 12 as well as by population group 10, 11, 13. It remains unknown whether the incidence of these malignancies in South Africa follows similar patterns to those reported in higher income areas. Recently, it was reported that the incidence of pediatric hematologic malignancies was approximately three times higher among White compared with Black children within South Africa 14. As population group is highly correlated with socioeconomic position and access to private health care services in South Africa 15, the authors hypothesized that differences in access and utilization of health care services likely explain at least some of the observed incidence differences 14. To our knowledge, these patterns have not been investigated among adults in South Africa.

In this report, we analyze for the first time the incidence of adult cases of leukemia, multiple myeloma and related diseases, HL, and NHL reported to the National Cancer Registry of South Africa (NCR) between 2000 and 2006, by age, gender, and population group.

## Material and Methods

### National cancer registry

A detailed description of the NCR has been published elsewhere 16. Briefly, the NCR (www.ncr.ac.za) is a pathology‐based registry, reporting on malignancies confirmed in public and private laboratories throughout the country. The registry includes only incident, primary invasive cancers based on confirmation by histology, cytology, or hematology. Trained coders at the NCR code the diagnoses from pathology reports based on primary site and morphological type according to the International Classification of Diseases for Oncology, third edition (ICD‐O‐3) 17. For 2000–2004, the NCR determined cancer diagnosis from pathologist reporting of SNOMED codes, rather than full pathology reports. For 2005–2006, full pathology reports were received from public laboratories but not private. Until 2011, reporting to the registry was done on a voluntary basis although all of the National Health Laboratory Services (NHLS) laboratories regularly reported to the registry as the NCR is a division of the NHLS. Reporting has been less complete from the private sector, particularly from 2005 onwards 16. In addition to basic demographic information about the patient (name, age and/or date of birth, gender) and tumor diagnosis information (topography, morphology, date of diagnosis), the registry extracts information on population group (Black, White, Coloured (i.e., mixed ancestry), and Asian/Indian), where available from the pathology reports. If not found on the pathology report, a hot‐deck imputation method is used to estimate population group using a database of approximately 1.4 million surnames with known population group 16. In the dataset used for this analysis, 66.8% of the case reports had missing population group and thus a substantial proportion was imputed. If population group cannot be estimated (i.e., surname with no match in the database), it is left as missing. A comparison of the distribution of population group based on actual versus imputed data for a subset of 277130 cancer cases (contributing to the database) reported to the registry between 1990 and 1995 showed very good agreement between actual and imputed values. The distribution of original versus imputed () data was as follows: 53% (50%) White, 40% (41%) Black, 2% (2%) Asian/Indian, and 5% (7%) Coloured (chi‐squared test *P*‐value = 0.94 for distribution differences).

### Hematologic malignancy cases

For the present report, we included all pathology‐confirmed incident cases of leukemia, myeloma and related diseases (subsequently referred to as myeloma), HL, and NHL reported to the NCR that were diagnosed at ages ≥15 years between 2000 and 2006. We followed the Surveillance, Epidemiology and End Results (SEER) Program site recode (http://seer.cancer.gov/siterecode/icdo3_dwhoheme/index.html) which is based on the ICD‐O‐3 17 and the 2008 WHO Classification of Tumours of Haematopoietic and Lymphoid Tissues 18 for classification of these four groups (see Tables 1 and 2 for listing of ICD‐O‐3 codes). The lack of full pathology reports prevented implementation of the more detailed classification of lymphoid neopslasms 18. Our analysis did not include hematologic malignancies other than leukemia, myeloma, HL, or NHL.

**Table 1:** Distribution of pathology‐confirmed hematologic malignancies^a^ reported to National Cancer Registry in South Africa by select characteristics at age ≥15 years for 2000–2006

	Females											Males										
	Black		White		Coloured		Asian/Indian		Unknown		All	Black		White		Coloured		Asian/Indian		Unknown		All
	*N*	%	*N*	%	*N*	%	*N*	%	*N*	%	*N*	*N*	%	*N*	%	*N*	%	*N*	%	*N*	%	*N*
Year of diagnosis																						
2000	458	50	278	30	113	12	38	4	27	3	914	563	50	365	32	126	11	37	3	44	4	1135
2001	482	50	338	35	77	8	43	4	26	3	966	563	49	397	35	102	9	40	4	38	3	1140
2002	496	52	317	33	77	8	33	3	30	3	953	542	49	365	33	99	9	57	5	49	4	1112
2003	413	48	287	33	97	11	34	4	29	3	860	518	48	376	35	107	10	37	3	36	3	1074
2004	514	49	332	32	116	11	34	3	55	5	1051	500	46	366	34	127	12	36	3	60	6	1089
2005	499	51	296	30	96	10	31	3	64	6	986	466	47	326	33	115	11	32	3	63	6	1002
2006	585	55	296	28	110	10	25	2	56	5	1072	635	50	383	30	141	11	35	3	68	5	1262
Total	3447	51	2144	32	686	10	238	3	287	4	6802	3787	48	2578	33	817	10	274	4	358	5	7814
Reporting source																						
NHLS	2937	60	1063	22	531	11	166	3	171	4	4868	3101	57	1294	24	618	11	184	3	201	4	5398
Private	510	26	1081	56	155	8	72	4	116	6	1934	686	28	1284	53	199	8	90	4	157	6	2416
Total	3447	51	2144	32	686	10	238	3	287	4	6802	3787	48	2578	33	817	10	274	4	358	5	7814
Age, years																						
15–19	170	64	51	19	29	11	7	3	8	3	265	237	64	67	18	34	9	20	5	15	4	373
20–24	181	60	58	19	32	11	19	6	11	4	301	225	56	82	21	48	12	26	7	19	5	400
25–29	337	73	68	15	24	5	17	4	16	3	462	266	65	83	20	28	7	11	3	19	5	407
30–34	429	73	75	13	37	6	14	2	29	5	584	469	69	82	12	62	9	23	3	41	6	677
35–39	361	67	91	17	43	8	16	3	26	5	537	414	63	137	21	56	8	17	3	35	5	659
40–44	341	64	91	17	61	11	20	4	24	4	537	422	59	156	22	77	11	25	4	30	4	710
45–49	340	59	138	24	51	9	24	4	27	5	580	410	58	167	23	71	10	34	5	30	4	712
50–54	303	50	185	30	65	11	31	5	27	4	611	383	48	253	32	96	12	28	4	34	4	794
55–59	263	42	244	39	70	11	24	4	21	3	622	290	42	271	39	85	12	23	3	29	4	698
60–64	238	37	266	42	87	14	20	3	28	4	639	231	33	310	45	81	12	30	4	40	6	692
65–69	164	31	241	46	67	13	19	4	30	6	521	175	29	312	51	86	14	14	2	24	4	611
70–74	157	33	241	50	51	11	14	3	17	4	480	137	28	279	57	48	10	12	2	17	3	493
75–79	91	26	211	59	28	8	11	3	14	4	355	79	23	219	63	31	9	6	2	12	3	347
80+	72	23	184	60	41	13	2	1	9	3	308	49	20	160	66	14	6	5	2	13	5	241
Total	3447	51	2144	32	686	10	238	3	287	4	6802	3787	48	2578	33	817	10	274	4	358	5	7814

a ICD‐O‐3 morphology codes 9590‐9999 excluding 9741, 9750, 9755, 9756, 9757, 9758, 9761, 9950, 9960, 9961, 9962, 9980, 9982, 9983, 9989 (153 cases excluded). NHLS, National Health Laboratory Systems.

**Table 2:** Crude and age‐standardized rates per 100,000 persons of pathology‐confirmed hematologic malignancies reported to NCR‐SA per 100,000 by race and gender at age ≥15 years for 2000–2006

Gender	Hematologic malignancy	Black			White			Coloured			Asian/Indian			All^a^		
		*N*	Crude	ASR_15_+	*N*	Crude	ASR_15_+	*N*	Crude	ASR_15_+	*N*	Crude	ASR_15_+	*N*	Crude	ASR_15_+
Females	Leukemia^b^	758	0.85	0.97	390	2.78	2.39	123	1.20	1.40	59	1,84	1,84	1394	1.19	1.32
	Myeloma^c^	456	0.51	0.68	191	1.36	1.06	94	0.89	1.23	15	0,47	0,55	788	0.67	0.83
	HL^d^	366	0.41	0.42	229	1.63	1.69	85	0.80	0.82	44	1,38	1,36	749	0.64	0.65
	NHL^e^	1867	2.09	2.26	1334	9.50	7.60	384	3.63	4.60	120	3,75	4,06	3871	3.30	3.65
Males	Leukemia^b^	864	1.06	1.32	568	4.32	3.86	162	1.70	2.26	71	2,31	2,40	1737	1.62	2.01
	Myeloma^c^	432	0.53	0.83	196	1.49	1.23	77	0.81	1.24	17	0,55	0,72	755	0.70	1.00
	HL^d^	516	0.63	0.63	305	2.32	2.32	114	1.20	1.22	61	1,99	2,10	1041	0.97	0.99
	NHL^e^	1975	2.43	2.89	1509	11.48	9.85	464	4.87	6.57	125	4,07	4,69	4281	4.00	4.98

ASR_15_+ – age‐standardized rates, truncated to ages ≥15 years. HL, Hodgkin lymphoma; NHL, Non‐Hodgkin lymphoma; ICD‐O‐3, International Classification of Diseases for Oncology, third edition.

a Includes cases with unknown population group.

b Leukemia – ICD‐O‐3 morphology codes: 9826, 9835–9836, 9820, 9832–9834, 9940, 9840, 9861, 9865–9867, 9869, 9871–9874, 9895–9897, 9898, 9910–9911, 9920, 9891, 9863, 9875–9876, 9945–9946, 9860, 9930, 9801, 9805–9809, 9931, 9733, 9742, 9800, 9831, 9870, 9948, 9963–9964. Also includes 9811–9818, 9837, 9823, and 9827 if site code C42.0, C42.1 or C42.4

c Myeloma – ICD‐O‐3 morphology codes: 9731–9732, 9734.

d Hodgkin lymphoma – ICD‐O‐3 morphology codes: 9650–9667.

e Non‐Hodgkin lymphoma – ICD‐O‐3 morphology codes: 9590–9597, 9670–9671, 9673, 9675, 9678–9680, 9684, 9687–9691, 9695, 9698–9702, 9705, 9708–9709, 9712, 9714–9719, 9724–9729, 9735, 9737–9738. For sites other than C42.0, C42.1, and C42.4 also includes 9811–9818, 9823, 9827, 9837.

### Population data

Consistent with the approach used in the annual reports of the NCR for the years included in this study, we used the alternative South African mid‐year population estimates 19 from the Centre for Actuarial Research, University of Cape Town, stratified by age, gender, and population group for calculation of incidence rates. These mid‐year population estimates are similar in magnitude to the official mid‐year estimates, but maintain an age distribution that is consistent with that of the most recent census in 2011 and, as with the NCR for this time period, we considered them as the more appropriate population estimates for the purposes of estimating age‐specific and age‐standardized rates.

### Statistical methods

Gender‐specific crude incidence rates overall and stratified by population group were estimated for leukemia, myeloma, HL, and NHL. Reflecting limited case numbers in individual age groups, age‐specific rates were not estimated separately for males and females. Gender‐specific age‐standardized rates, overall and stratified by population group, were calculated using the SEGI world standard 20 truncated for ages ≥15 (ASR_15_^+^). The ASR_15_^+^ is a weighted average of age‐specific rates based with the following weights for each age group: 15–19 (0.13), 20–24 (0.12), 25–29 (0.12), 30–34 (0.09), 35–39 (0.09), 40–44 (0.09), 45–49 (0.09), 50–54 (0.07), 55–59 (0.06), 60–64 (0.06), 65–69 (0.04), 70–74 (0.03), 75–79 (0.01), and 80+ (0.01).

Incidence rate ratios (IRRs) and 95% confidence intervals (CIs) were estimated using Poisson regression models with the number of cases for a given category of hematologic malignancy as the outcome, the population size as the log offset and a log link function, overall and stratified by population group, comparing rates among females to males. Similar models, overall and stratified by gender, were used to compare incidence rates by population group, using the Black population group as the reference group. All models were adjusted for age group (5 year categories) and calendar year (single‐year treated as a categorical variable). Models including all population groups and/or both males and females were further adjusted for population group and gender. Hematologic patients with unknown population group and/or gender (4.7%) were excluded from Poisson models as there were no corresponding population estimates for such groups. As under‐ascertainment of cancers may be more prominent at older ages (in many settings)^21^, sensitivity analyses were repeated by restricting the dataset to ages <75 years.

## Results

Between 2000 and 2006, there were a total of 14662 cases of leukemia, myeloma, HL, and NHL reported to the registry. There were 46 cases (0.3%) with unknown gender. Table 1 presents the distribution by population group for the 14616 cases with known gender, by calendar year of diagnosis, reporting source (private vs. public), and age at diagnosis, separately for males and females. In all calendar years, approximately half of the cases were reported among the Black, 33% among the White, 10% among the Coloured, and 5% or less among the Asians/Indian population groups. The distribution of population group differed substantially between public and private laboratories, with the White population group accounting for approximately half of all cases reported by private laboratories. With increasing age at diagnosis, the proportion of cases coming from the Black population group declined while that from the White population group increased steadily. Similar patterns were observed for males and females with respect to calendar year, reporting source and age.

**Figure 1:**
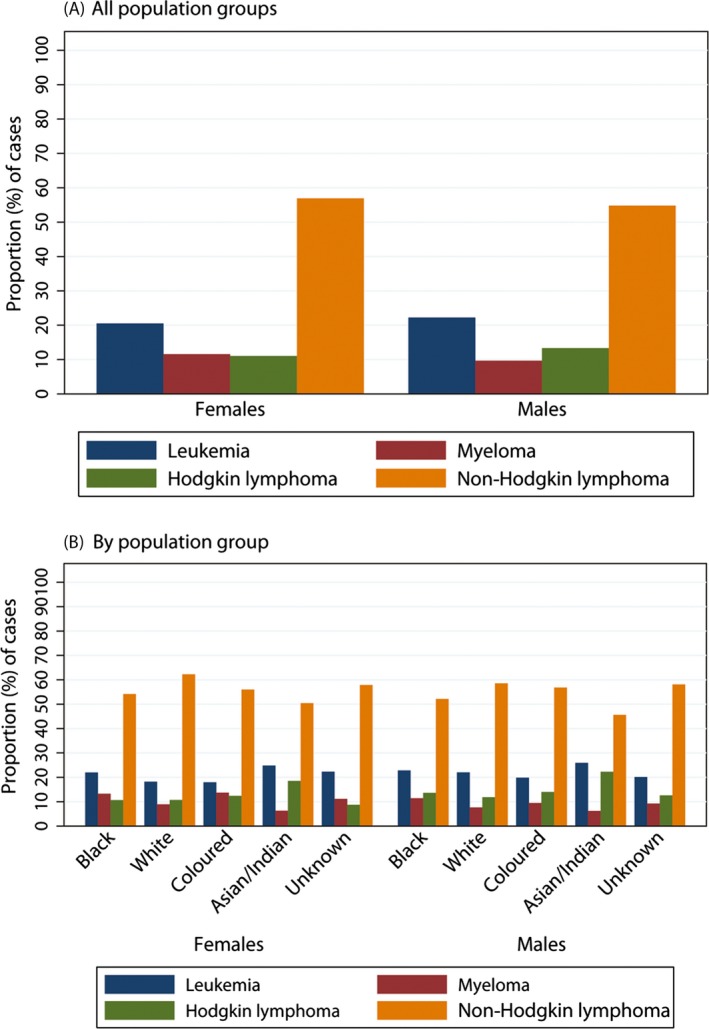
Distribution of pathology‐confirmed cases of hematologic malignancies reported to the National Cancer Registry of South Africa in 2000–2006 at age ≥15 years for (A) all population groups combined and (B) by population group.

The breakdown of hematologic malignancies types is presented for males and females separately, overall and by population group, in Figure 1. Regardless of gender or population group, NHL was the most commonly reported hematologic malignancy, accounting for approximately 50% or more of cases in most population groups (Fig. 1B). In all groups, this was followed by leukemia, contributing 15–25% of cases in the various subgroups.

Crude and age‐standardized incidence rates are presented for leukemia, myeloma, HL, and NHL by population group and gender in Table 2. Incidence rates varied markedly by population group; in general, the lowest rates were observed among the Black population group and the highest among the White population group. An exception was myeloma, for which rates were lowest among the Asian/Indian population group for both males and females.

**Figure 2:**
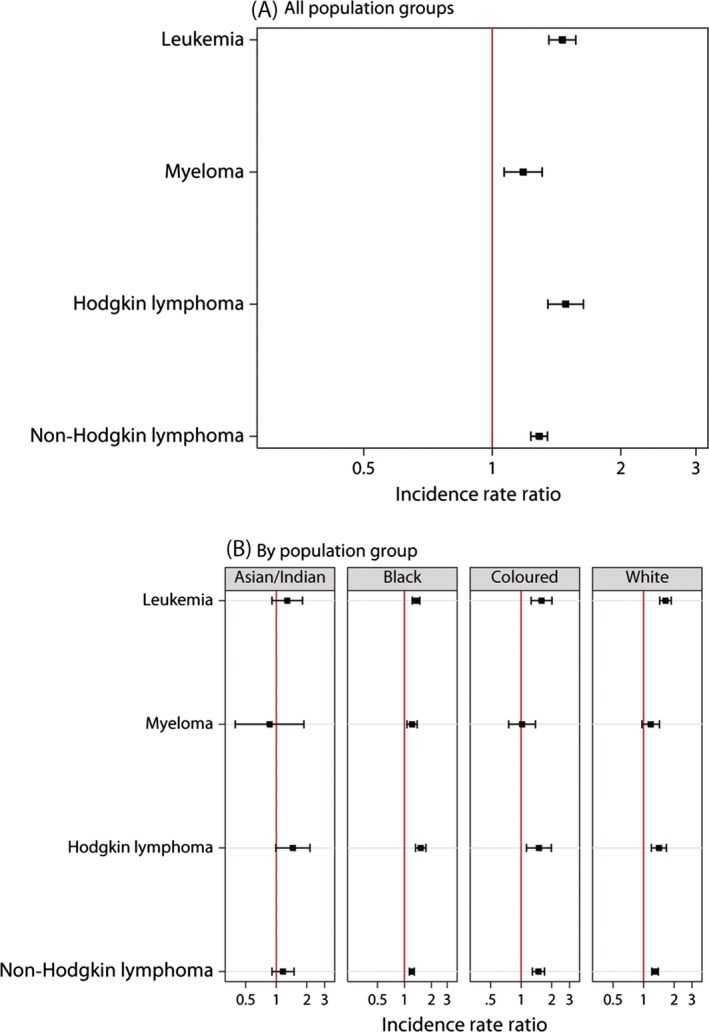
Incidence rate ratios of pathology‐confirmed hematologic malignancies reported to the National Cancer Registry of South Africa in 2000–2006 comparing males to females at age ≥15 years for (A) all population groups combined and (B) by population group. Incidence rate ratios estimated from Poisson regression model adjusted for age (5 year categories), calendar year (1 year categories) and population group. Cases with missing gender or population group excluded from analyses.

Figure 2 presents the IRR comparing males to females. For all population groups combined, the reported incidence rate of hematologic malignancies was 1.2 to 1.5‐fold higher among males than females (Figure 2a). Similar patterns were observed across the four population groups (Figure 2b).

**Figure 3:**
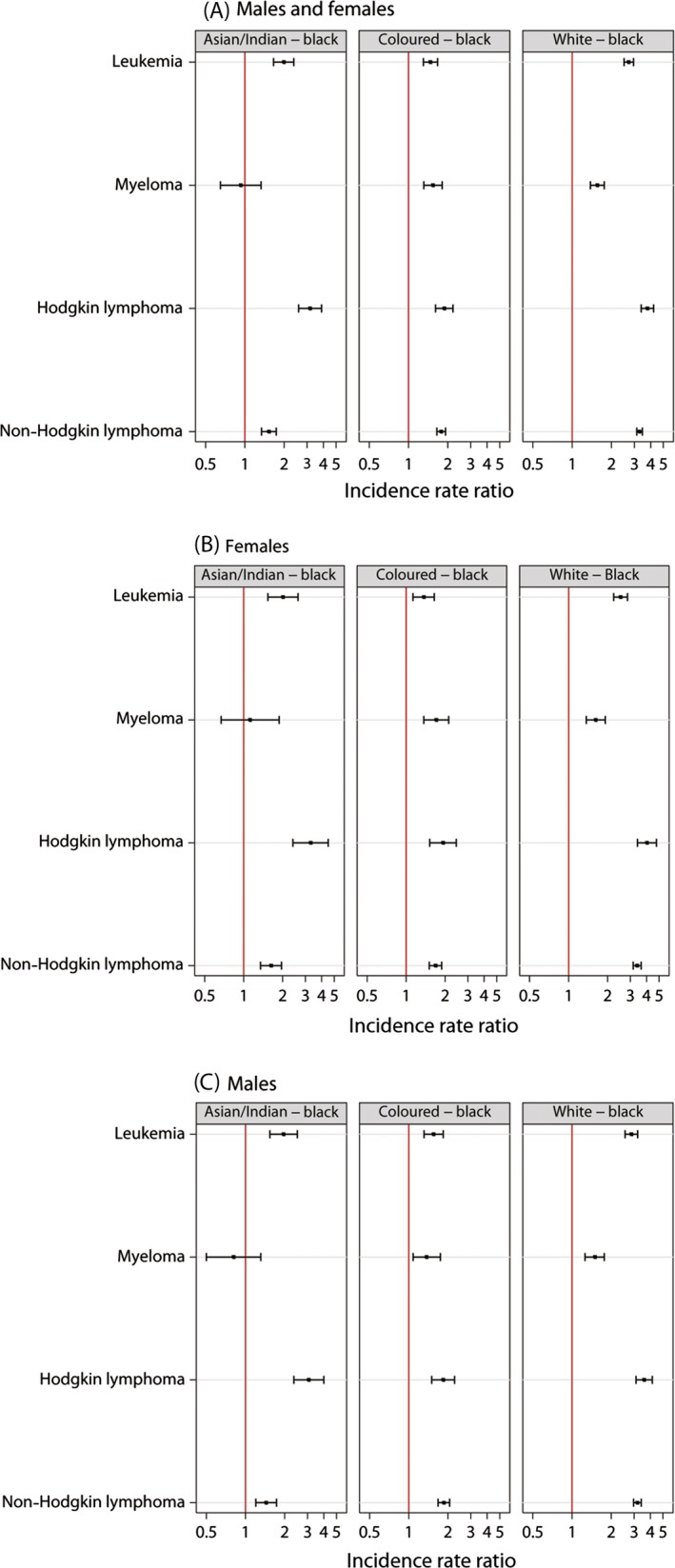
Incidence rate ratios of pathology‐confirmed hematologic malignancies reported to the National Cancer Registry of South Africa in 2000–2006 comparing the Asian/Indian, Coloured and White population groups to the Black population group at age ≥15 years for (A) males and females combined, (B) females, and (C) males. Incidence rate ratios estimated from Poisson regression model adjusted for age (5 year categories), calendar year of diagnosis (1 year categories) and gender. Cases with missing gender or population group excluded from analyses.

The IRR for population group are presented in Figure 3. For males and females combined, reported incidence rates of hematologic malignancies tended to be higher among the White, Coloured, and Asian/Indian population groups than among the Black population group (Fig. 3A). The exception was for myeloma, for which no statistically significant difference was observed between the Asian/Indian and Black population groups, in either males or females. The largest rate ratios were observed comparing the White and Black population groups, ranging from 1.56 (95% CI 1.38–1.76) for myeloma to 3.77 (95% CI 3.38–4.21) for HL. Gender‐specific patterns were similar to those observed for males and females combined (Fig. 3B and C).

**Figure 4:**
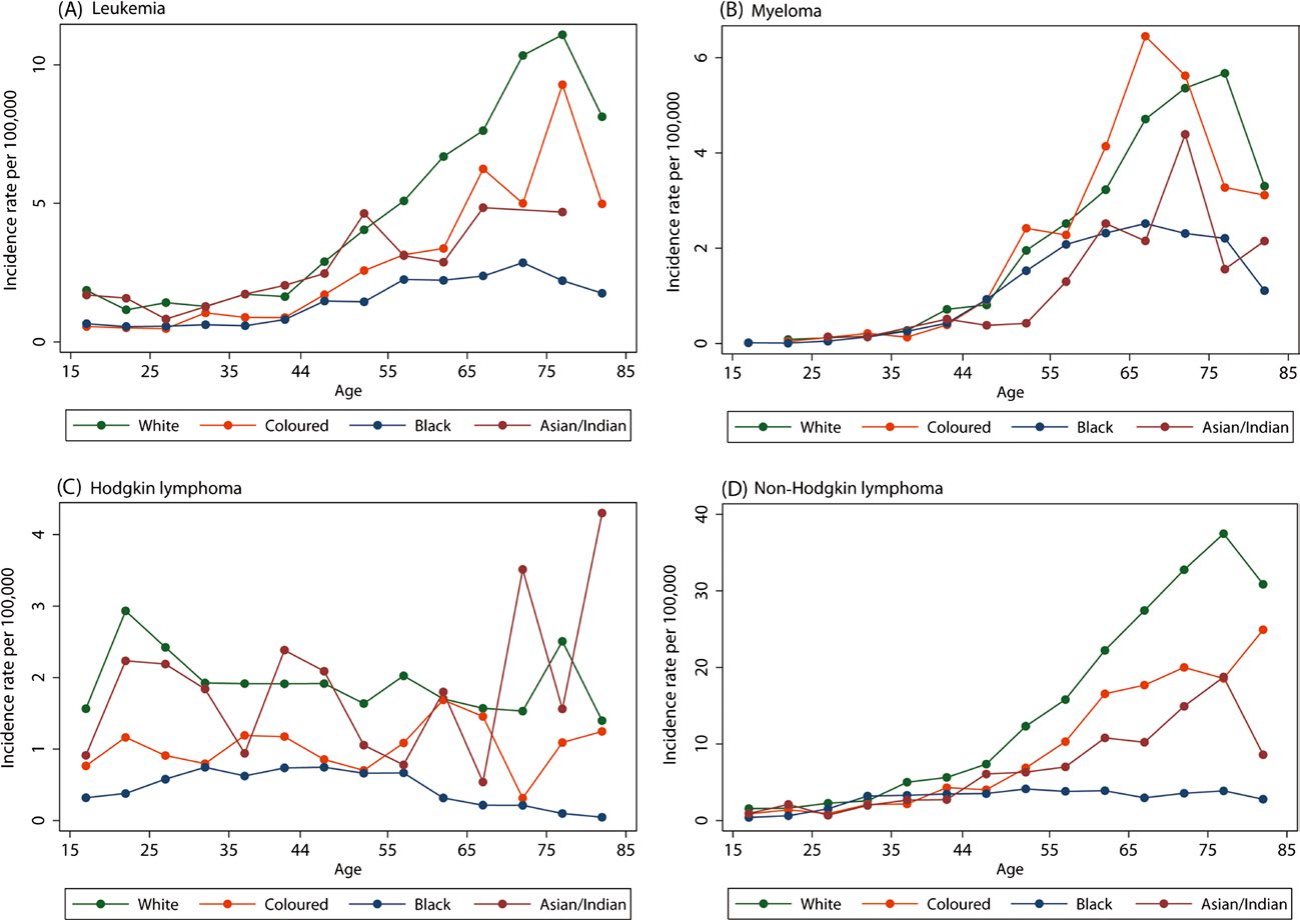
Age‐specific rates (per 100,000) of pathology‐confirmed (A) leukemia, (B) myeloma, (C) Hodgkin lymphoma, and (D) Non‐Hodgkin lymphoma reported to the National Cancer Registry of South Africa in 2000–2006 by population group at age ≥15 years, males and females combined. Cases with missing gender or population group excluded from analyses.

Age‐specific rates of leukemia, myeloma, HL, and NHL are presented in Figure 4A–D by population group. With the exception of HL, incidence rates tended to increase with age until approximately age 75, followed by a decline at the oldest ages. For HL (Fig. 4C), the patterns appeared quite different between the population groups, most notably comparing the White and Black population groups. Among the White population group, there was an early peak in HL incidence rates at ages 20–29 and a later peak around age 70–75 with rates somewhat lower and generally stable in between these age groups. Among the Black population group, there was an increase with HL with age until approximately age 30, at which point the rate plateaued followed by a subsequent decline beginning around age 60. For all four major types of hematologic malignancies investigated, incidence rates were consistently higher among the White population group than the Black population group, irrespective of age, but these differences tended to increase with age (Fig. 4A–D).

In sensitivity analyses restricted to ages <75, there were no marked changes in the results presented in Tables 1, 2 or Figures 1, 2, 3. Incidence rate ratios (IRRs) by population group were slightly attenuated at ages <75 compared with the full adult population, but the reduction was very minor and the interpretation unchanged. This observation is consistent with the patterns observed in age‐specific rates whereby differences between the White and Black population groups were most apparent at older ages.

## Discussion

### Summary of key results

The incidence of adult hematologic malignancies (diagnosed at ages 15 years or older) was estimated for laboratory‐confirmed cases reported to the NCR between 2000 and 2006, describing overall rates as well as those by age, gender, and population group. NHL was the most common hematologic malignancy reported to the NCR during this time period, irrespective of gender and population group. Incidence rates of reported hematologic malignancies were generally 20–50% higher among males than females. Our analyses suggested lower rates of reported hematologic malignancies among the Black population group compared with other population groups, with differences most pronounced when comparing the White and Black population groups. These differences tended to become more marked with increasing age. With respect to age‐specific rates, incidence rates increased with age for hematologic malignancies other than HL. For HL, among the White population group, a bimodal peak was observed at ages 20–29 and 70–75. A different pattern was observed among the Black population group; reported HL rates increased with age until approximately age 30, at which point the rate plateaued, followed by a subsequent decline beginning around age 60.

### Interpretation of key results

The observation that NHL, followed by leukemia, was the most common of these four broad categories of hematologic malignancies is consistent with worldwide patterns 1. The higher incidence rates among males than females are also consistent with gender patterns reported elsewhere 12, 22.

With respect to population group, age‐adjusted incidence rates from the U.S. Surveillance, Epidemiology and End Results (SEER) 18 registries in the United States for the period of 2000–2011 show a predominance among the White vs. Black populations with annual White to Black ratios (estimated using the SEER Fast Stats tool 23) of 1.3–1.5 for NHL, 1.2–1.4 for leukemia and 1.1–1.3 for HL. These estimates are somewhat lower than those estimated in the NCR data. Of note, previous analyses of the SEER data have shown that the magnitude and direction of these population group incidence rate ratios varies by subtype of leukemia and lymphoma 10, 11. As we did not have full pathology reports for all cases (as discussed in the Methods) we were not able to consider subtypes in this analysis. The apparently distinct age‐specific patterns observed for HL between the White and Black population groups in the NCR data are also reported in the SEER data where a clear bimodal pattern, classically associated with HL, is much more pronounced in the White than Black populations 24. Globally, the classic bimodal age pattern appears to be more a characteristic of more economically developed areas 25. In contrast to what is observed for NHL, HL, and leukemia rates, the incidence of myeloma in the SEER data is approximately twofold greater among the Black than White population groups 13. In contrast, the reported incidence rate of myeloma in the NCR was approximately 50% greater among the White than Black population groups.

For any cancer site, differences in the underlying distribution of genetic and environmental risk factors as well as factors related to completeness of reporting and diagnosis drive demographic variations in the incidence patterns. The etiology of hematologic malignancies is largely unexplained, with few known determinants 25, 26, 27. Established environmental risk factors for leukemia include ionizing radiation and certain chemical exposures such as benzene 27. For NHL, there is clear evidence for an association with infectious diseases (HIV, Epstein–Barr virus (EBV), Hepatitis C Virus (HCV), and Human T‐Lymphotrophic Virus (HTLV‐1)) 26 and increasing data to support a role for lifestyle, occupational, and environmental factors 28. HL also has an infectious etiology – EBV is one of few known risk factors 25. As is common for registry‐based studies, we did not have individual‐level information about risk factors. While we cannot exclude the possibility that differences in the distribution of or susceptibility to etiologic factors could explain the marked differences by population group observed in the NCR data, the known infectious and environmental risk factors would not seem likely explanations. In order for these factors to drive truly higher rates of disease within South African White versus Black population groups, they would need to be more prevalent in the White population group.

Disparities in the completeness of diagnosis and reporting between population groups may have contributed to the observed incidence rate patterns. The NCR is a pathology‐based registry and thus only hematologic malignancies with a histologic, cytologic, or hematologic (bone marrow aspirate or trephine biopsy) confirmation are captured. Consequently, there is an inherent risk of under‐estimating rates based on the registry data as cases diagnosed by other means (i.e., peripheral blood smear) are not reported. Problems of under‐reporting may be compounded, however, by other factors that disproportionately affect the Black population group compared with the White population group and contribute not only to under‐reporting but also under‐diagnosis of these cancers. First, a smaller proportion of the Black population group have access to a private medical aid fund (7.2% of the Black population versus 63.1% of the White population according to 2006 data) 15. Public medical services are chronically under‐resourced and understaffed 29, 30 and as such, patients in the public sector may be less likely than those in the private sector to receive a comprehensive diagnostic work‐up. Furthermore, the system operates under a tiered structure by which patients are referred from primary health clinics to tertiary hospitals via other tiers 31 and patients may be lost from the system before presenting at referral centers. Factors such as distance to the nearest tertiary center 32 may lead to delayed or no diagnosis. Second, the burden of HIV varies markedly by population group 33. While HIV is associated with increased risk of lymphomas, particularly subtypes of NHL 9, atypical presentation and histology of HIV‐associated lymphomas may lead to misdiagnosis or delayed diagnosis 34. In population groups with high HIV rates, competing mortality from other causes 9, 35 may reduce the opportunity for lymphomas to develop while late‐stage presentation of disease 3, 36 may lower the chances of cancers ever being diagnosed by pathology.

### Strengths/limitations

This is the first country‐wide study on hematologic malignancies in South Africa and one of very few studies from Sub‐Saharan Africa. The study benefits from the large number of cases permitting detailed examination of rates and patterns by age, gender, and population group. The diverse population of South Africa enabled us to investigate differences by population group which is associated with socioeconomic circumstances and access to private health care in South Africa 15. Nonetheless, analyses of the Asian/Indian and Coloured population groups were less robust owing to smaller case numbers than in the Black and White population groups, particularly when further examining age‐specific patterns. Limitations include that, by definition, the NCR data being used is restricted to pathology‐confirmed cancer cases and thus it is understood that it does not fully capture incident hematologic malignancies in the country. Furthermore, there was a decline in reporting to the NCR by some private sector laboratories (beginning in 2005) 16. This would be expected to have the greatest impact on cases reported from the White population group which could attenuate the observed rate ratios by population group. As discussed above, we were unable to implement more detailed subtype classification as full pathology reports were not available for the period of 2000–2004. Previous studies of lymphoma and leukemia in the United States suggest that racial differences may vary considerably by subtype 10, 11]. More generally, there are known etiologic differences in the subtypes of leukemia and lymphomas 26, 27, 28. Once available, it will be important to repeat these analyses using data from more recent years during which full pathology reports were received by the NCR. Another limitation is that population group had to be imputed for a substantial proportion of the dataset. The imputation method, however, has been previously validated in the NCR and the limitation appears to be of minor importance, although some misclassification cannot be ruled out. While it is important to consider our results in the context of these limitations, the NCR provides the most comprehensive overview of these cancers in the country at this time.

## Conclusions

The hematologic malignancies investigated here collectively account for an estimated 6% of new cancer cases and 8% of cancer deaths in South Africa 1. The consistency of patterns by age and gender with those reported in other populations 1, 10, 12, 13, 22, 24 suggest that underlying risk factors for these cancers are unlikely to modify the age distribution or gender ratio. Differences between population groups, however, would appear to be more pronounced than those observed in some other settings. We hypothesize that challenges related to diagnosis and reporting of cancers play a role in the patterns by population group while the set‐up of the NCR (pathology‐based) could lead to some degree of under‐ascertainment, irrespective of population group, gender, or age. Despite challenges, it is important to analyze and report available national cancer incidence data to raise awareness of the cancer burden and to characterize patterns by demographic characteristics so as ultimately to improve the provision of cancer diagnosis and care.

## Conflict of Interest

The authors have no conflicts of interest.
